# Long-term monitoring for short/branched-chain acyl-CoA dehydrogenase deficiency: A single-center 4-year experience and open issues

**DOI:** 10.3389/fped.2022.895921

**Published:** 2022-09-06

**Authors:** Alessandro Rossi, Mariagrazia Turturo, Lucia Albano, Simona Fecarotta, Ferdinando Barretta, Daniela Crisci, Giovanna Gallo, Rosa Perfetto, Fabiana Uomo, Fabiana Vallone, Guglielmo Villani, Pietro Strisciuglio, Giancarlo Parenti, Giulia Frisso, Margherita Ruoppolo

**Affiliations:** ^1^Department of Translational Medicine, Section of Pediatrics, University of Naples “Federico II”, Naples, Italy; ^2^Department of Molecular Medicine and Medical Biotechnology, University of Naples “Federico II”, Naples, Italy; ^3^CEINGE Biotecnologie Avanzate s.c.ar.l, Naples, Italy

**Keywords:** amino acid, 2-methylbutyrylglycinuria, biomarkers, monitoring, treatment, carnitine, diet

## Abstract

**Introduction:**

Short/branched-chain acyl-CoA dehydrogenase deficiency (SBCADD) is an inherited disorder of L-isoleucine metabolism due to mutations in the *ACADSB* gene. The role of current diagnostic biomarkers [i.e., blood 2-methylbutyrylcarnitine (C5) and urine 2-methylbutyrylglycine (2MBG)] in patient monitoring and the effects of proposed treatments remain uncertain as follow-data are lacking. This study presents first systematic longitudinal biochemical assessment in SBCADD patients.

**Methods:**

A retrospective, observational single-center study was conducted on newborns born between 2017 and 2020 and suspected with SBCADD. Biochemical, molecular, clinical and dietary data collected upon NBS recall and during the subsequent follow-up were recorded.

**Results:**

All enrolled subjects (*n* = 10) received adequate protein intake and L-carnitine supplementation. Nine subjects were diagnosed with SBCADD. During the follow-up [median: 20.5 (4–40) months] no patient developed symptoms related to SBCADD. No patient normalized serum C5 and urine 2MBG values. In 7/9 SBCADD patients mean serum C5 values decreased or stabilized compared to their first serum C5 value. A major increase in serum C5 values was observed in two patients after L-carnitine discontinuation and during intercurrent illness, respectively. Urine 2MBG values showed moderate intra-patient variability.

**Discussion:**

The relatively stable serum C5 values observed during L-carnitine supplementation together with C5 increase occurring upon L-carnitine discontinuation/intercurrent illness may support the value of serum C5 as a monitoring biomarker and the benefit of this treatment in SBCADD patients. The role of urine 2MBG in patient monitoring remains uncertain. As all patients were asymptomatic, no association between biochemical parameters and clinical phenotype could be investigated in this study.

## Introduction

Short/branched-chain acyl-CoA dehydrogenase (SBCAD) deficiency (SBCADD) (OMIM# 600301, also known as 2-methylbutyrylglycinuria, OMIM#610006) is an autosomal recessive metabolic disorder due to mutations in the *ACADSB* gene (1, 2). This gene encodes for the mitochondrial SBCAD, which catalyzes (1) the third reaction in the degradation pathway of L-isoleucine (that is the conversion of 2-methylbutyryl-CoA to tiglyl-CoA) and (2) the first oxidative step of short straight-chain acyl-CoAs (such as butyryl-CoA and hexanoyl-CoA). SBCAD could also use valproyl-CoA as a substrate, potentially playing a role in valproate metabolism (3, 4).

Most individuals with SBCADD, including those ascertained *via* NBS, show no health problems (5). However, a small percentage of individuals can develop signs and symptoms soon after birth or later in childhood, including poor feeding, lethargy, vomiting, irritability (6, 7). These symptoms can eventually progress to serious conditions such as dyspnea, seizures, and coma (5, 7). Additional features include poor growth, muscle weakness, delay in motor skills (6, 8–10), intellectual disability (11).

SBCADD is associated with elevated 2-methylbutyryl carnitine (C5) and 2-methylbutyrylglycine (2MBG) concentrations in blood and urine, respectively. Since C5 is detectable by tandem mass spectrometry (MS/MS) on dried blood spots (DBS) (and serum), SBCADD may be identified through expanded newborn screening (NBS) (5, 12, 13). Assessment of urine 2MBG represents (one of) the major confirmatory tests (3).

Despite the progress in patient ascertainment, multiple uncertainties on the management of SBCADD still exist (13, 14). Particularly, the role of currently employed diagnostic biomarkers (i.e., blood C5 and urine 2MBG) in patient monitoring is unknown as follow-up data are largely missing. In addition, there are no conclusive data on the efficacy of proposed treatments, namely L-carnitine supplementation and dietary protein restriction (15). With the gradual development of NBS programs worldwide, the number of individuals diagnosed with SBCADD as well as the phenotype variability is expected to increase over next years (16). Thus, it is important to define appropriate patient monitoring and management options.

The aim of the current study was to define the applicability of currently employed diagnostic biomarkers as monitoring tools by presenting the first systematic longitudinal biochemical assessment in SBCADD patients.

## Methods

### Subjects

This was a retrospective, observational single center study conducted at the Regional NBS reference center Campania, Italy, where the NBS samples of all newborns born in Campania Region are processed. Collectively, 160,015 subjects were analyzed in the study period. Subjects were enrolled if they met all the following inclusion criteria: (I) date of birth from 01 January 2017 to 31 December 2020; (II) increased C5 value (reference value 0.02–0.26 μmol/L) found on the NBS DBS sample; (III) increased urine 2MBG value (reference value <2 mmol/mol Creatinine) found upon NBS recall. Exclusion criteria were: (I) normal urine 2MBG value found upon NBS recall; and/or (II) detectable urine isovaleryglycine found upon NBS recall.

The Regional Operative Procedure for newborns suspected with an inherited metabolic disease at NBS is presented in Supplementary Figure 1. Briefly, newborns suspected with SBCADD are included in a follow-up program and undergo: (1) routine clinical and biochemical assessment, (2) molecular testing of the *ACADSB* gene, and (3) oral L-carnitine supplementation (100 mg/kg/day) initiated upon NBS recall. Additionally, all caregivers are instructed on avoiding prolonged fasting and checking blood glucose concentrations upon metabolic stress conditions (e.g., intercurrent illness, decreased oral intake).

### Methods

All enrolled subjects were clinically and biochemically evaluated upon NBS recall and subsequently every 3–6 months. Collected data were retrieved from patients' records compiled during routine visits and included: DBS or serum C5 concentration, urine 2MBG concentration, serum glucose, ammonia and CK concentrations, liver function tests, blood gases, heart ultrasound, electrocardiogram, weight, height, and psychomotor development assessment as well as information on medical history, daily protein intake, L-carnitine supplementation, and *ACADSB* variants.

C5 and 2MBG concentrations were assessed on morning samples (≥2-h fasting). C5 concentration was evaluated through acylcarnitine analysis. Acylcarnitine analysis was performed on DBS samples upon NBS recall and on serum samples during subsequent evaluations by tandem-mass spectrometry (LC/MS-MS) as previously described (17, 18). Urine 2MBG concentration was evaluated through urine organic acids (UOA) profile assessed by gas chromatography-mass spectrometry (GC-MS), as previously described (19). Additional biochemical parameters were evaluated by using routine assays with commercially available kits.

The intra-day precision was evaluated by analyzing three replicate analyses of three different serum and was estimated to be 0.7% coefficient of variation (CV). The inter-day precision was evaluated by analyzing three replicate analyses of three different serum over a 5-day period and was estimated to be 5%CV.

For each subject the serum C5 and urine 2MBG trend was defined as follows: (i) increased, if the difference between the first serum C5 value and the mean serum C5 value measured during the follow-up was > +10%, (ii) stable, if the difference between the first serum C5 value and the mean serum C5 value measured during the follow-up was between −10 and +10% and (iii) decreased, if the difference between the first serum C5 value and the mean serum C5 value measured during the follow-up was < −10%. The 10% threshold was chosen being around 10 times the intraday CV and twice the interday CV.

Molecular testing was performed on DNA extracted from EDTA peripheral venous blood samples. All exons and part of the flanking intron regions of *ACADSB* gene were amplified by polymerase chain reactions and sequenced for mutation analysis, according to standard procedure (20). Variations were reported following the Human Genome Variation Society (HGVS) nomenclature (http://www.HGVS.org/varnomen) and annotated according to NCBI SNPs Database (http://www.ncbi.nlm.nih.gov, accessed March 2022), ClinVar database (https://www.ncbi.nlm.nih.gov/clinvar, accessed March 2022), Human Gene Mutation Database (HGMD) Professional (http://www.hgmd.cf.ac.uk/, accessed March 2022), and American College of Medical Genetics and Genomics (ACMG) guidelines for variant classification (21).

## Results

Ten subjects were enrolled in the present study. Median follow up was 20.5 (range: 4–40) months. No abnormal findings in serum glucose, ammonia, CK, blood gases concentrations, liver function tests, heart ultrasound, electrocardiogram were detected in any of the subjects during the follow-up. All subjects showed regular growth and psychomotor development. No subjects received a protein restricted diet (median protein intake: 3.5 g/kg/day) (Table 1). Weaning occurred at 4–6 months in all subjects (median protein intake: 1.7 g/kg/day, range: 1.5–2.0 g/kg/day). All subjects were started with oral L-carnitine supplementation (100 mg/kg/day) upon NBS recall and continued the treatment for the whole duration of the follow-up, unless stated otherwise.

**Table 1:** Biochemical, clinical and molecular features of SBCADD patients.

**Subject**	**Initial C5 value^b^ (μmol/L)**	**Initial urine 2MBG^c^ (mmol/creatinine moles)**	**Follow up duration (months)**	**Clinical aspects**	**Daily Protein intake (g/Kg/day)^d^**	**Genotype** ***ACADSB* cDNA (protein variation)**	**Family segregation**	**Variant classification**			
								**Reference** **SNP ID**	**ClinVar**	HGMD*	ACMG**
P1	0.84	8	30	Asymptomatic	3.9	**c.641C>A (p.Ala214Glu)** c.1102T>C (p.Ser368Pro)	**F: c.641C>A** M: c.1102T>C	rs887880417 rs774205809	NR VUS	NR CM052826	**LP** VUS/LP
P2	0.57	20	24	Asymptomatic	3.5	c.1128+3A>T (possible splicing alteration) c.443C>T (p.Thr148Ile)	F: c.1128+3 A>T M: c.443C>T	rs760423996 rs58639322	CI CI	NR CM052824	LP P
P3^a^	0.68	8	40	Asymptomatic	3.4	c.1128+3A>T (possible splicing alteration) c.1159G>A (p.Glu387Lys)	F: c.1128+3A>T M: c.1159G>A	rs760423996 rs188094280	CI CI	NR CM080029	LP LP
P4^a^	0.54	11	40	Asymptomatic	3.4	c.1128+3A>T (possible splicing alteration) c.1159G>A (p.Glu387Lys)	F: c.1128+3A>T M: c.1159G>A	rs760423996 rs188094280	CI CI	NR CM080029	LP P
P5	0.68	60	17	Asymptomatic	3.8	c.443C>T (p.Thr148Ile) c.443C>T (p.Thr148Ile)	NP	rs58639322	CI	CM052824	P
P6	0.63	15	12	Asymptomatic	3.5	c.443C>T (p.Thr148Ile) c.1159G>A (p.Glu387Lys)	F: c.443C>T M: c.1159G>A	rs58639322 rs188094280	CI CI	CM052824 CM080029	P P
P7	0.69	20	4	Asymptomatic	3.5	c.908G>C (p.Gly303Ala) c.443C>T (p.Thr148Ile)	F: c.908G>C M: c.443C>T	rs1316417761 rs58639322	NR CI	CM052825 CM052824	LP P
P8	0.48	11	4	Asymptomatic	3.8	c.443C>T (p.Thr148Ile) **c.439A>T (p.Asn147Tyr)**	F: c.443C>T M: c.439A>T	rs58639322 rs747291865	CI NR	CM052824 NR	P **LP**
P9	0.43	22	30	Asymptomatic	3.2	**c.247A>G (p.Met83Val)** **c.293T>G (p.Phe98Cys)**	F: c.247A>G M: c.293T>G	rs751301851 NR	NR NR	NR NR	**LP** **LP**

C5 values detected on DBS samples and urine 2MBG values found upon NBS recall are shown. For each genetic variant the following information is shown: variant nomenclature at cDNA and protein level, according to the HGVS (Human Genome Variation Society) guidelines; reference single nucleotide polymorphism (SNP) ID number (rs); clinical significance by ClinVar database, HGMD database (*Human Genetic Mutation Database) and ACMG (**American College of Medical Genetics) classification, respectively. Novel variants are presented in bold. CI, conflicting interpretation; LP, likely pathogenic; NP, not performed; NR, not reported; P, pathogenic; VUS, variant of uncertain significance.

^a^Twin subjects.

^b^Reference value: 0.02–0.26 μmol/l.

^c^Reference value: <2 mmol/creatinine moles.

^d^Before weaning.

Nine subjects (P1–P9) showed homozygosity or compound heterozygosity for *ACADSB* variants and were diagnosed with SBCADD (Table 1). One subject (P10) only carried the heterozygous c.1159G>A (p.Glu387Lys) (reference SNP ID rs188094280) *ACADSB* variant. Although such variant is classified as pathogenetic according to the ACMG guidelines, this subject could not be conclusively diagnosed with SBCADD. Among the 9 detected variants, 4 were not previously described as associated to SBCADD, being missing in ClinVar and HGMD databases. According to the novel ACMG criteria, all variants were classified as (likely) pathogenic, [(L)P], except c.1102T>C, which was classified as VUS/likely pathogenic. However, this variant was previously reported in the HGMD database as associated with SBCADD patient (22).

**Figure 1:**
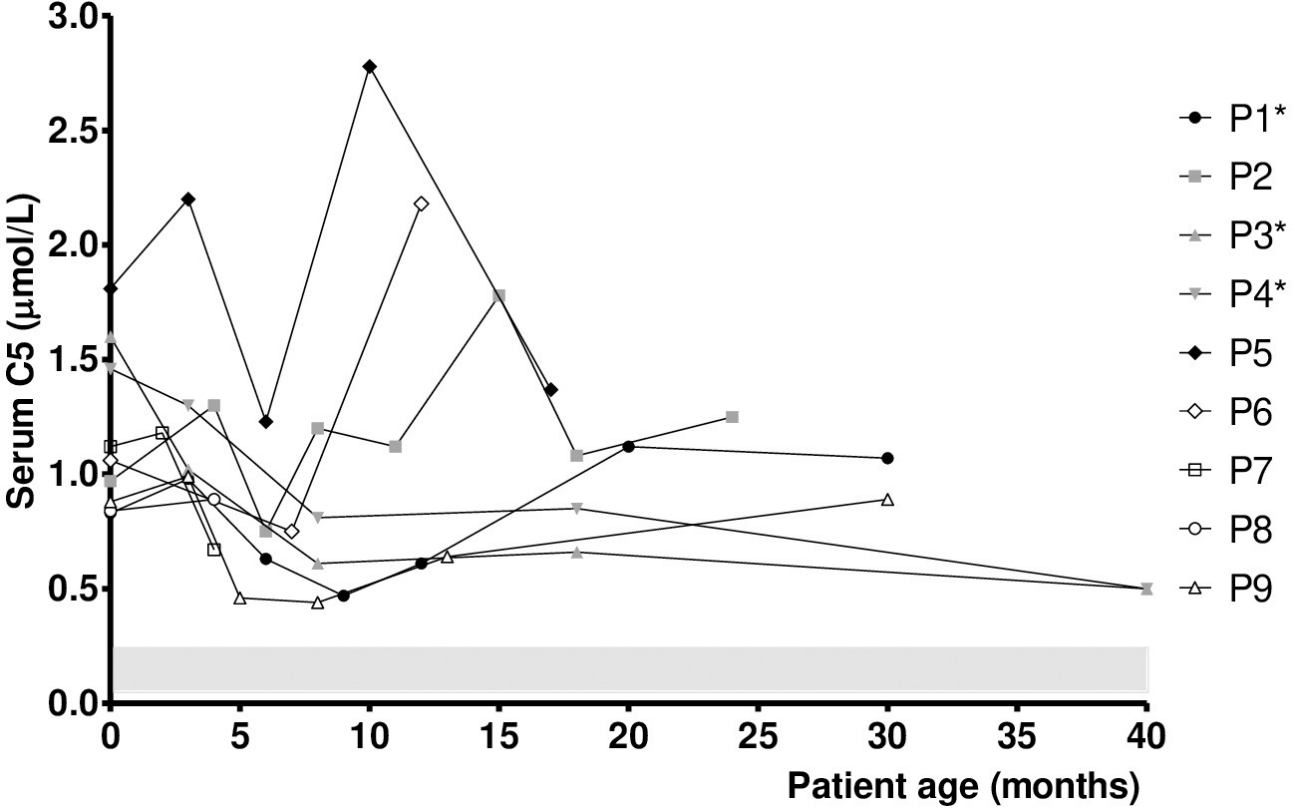
Serum C5 values in SBCADD patients. For each subject, the first time point shows the serum C5 value detected upon NBS recall before starting the L-carnitine treatment (100 mg/kg/day). Subsequent time points show serum C5 value after starting with L-carnitine treatment. C5 reference range is highlighted (shaded area). ^*^Data for P1 at 24 months, P3 and P4 at 12 and 24 months were not collected due to COVID19 pandemic.

The median DBS C5 and urine 2MBG values upon NBS recall in SBCADD patients were 0.63 μmol/l (reference value 0.02–0.26 μmol/l) and 15.0 mmol/creatinine moles (reference values <2 mmol/creatinine moles), respectively. Their median serum C5 and urine 2MBG values during follow-up were 1.0 μmol/l (reference values 0.05–0.24 μmol/l) and 21.0 mmol/creatinine moles (reference values <2 mmol/creatinine moles), respectively. Figure 1 presents the individual serum C5 values evaluated during the follow-up of SBCADD patients. Serum C5 values stayed above the reference values in all subjects. In 4/9 SBCADD patients (P3, P4, P7, P9) the individual serum C5 value decreased (range −20/−55%). In 3/9 SBCADD patients (P1, P5, P8) the individual serum C5 value stabilized (range −2/+6%). In 2/9 SBCADD patients (P2, P6) the individual serum C5 value increased (range +25/+39%). Serum C5 values showed a similar trend in subjects P3 and P4 (twin subjects). P5 showed the highest baseline serum C5 value among SBCADD patients. In this subject a major increase in serum C5 value was noted at 8 months, when an intercurrent illness (vomiting and diarrhea) without metabolic decompensation occurred. In P6 a major increase in serum C5 value occurred at 8 months. Tracing back the patient's history, it was ascertained that from this point the family had decided to discontinue L-carnitine supplementation because of its unpleasant taste. In P2 a transient increase in serum C5 value was noted at 3 and 14 months. At 3 months an intercurrent illness (vomiting, reduced food intake) occurred. No known factors possibly associated with the subsequent increase occurring at 14 months could be found. In P9 oral L-carnitine supplementation was discontinued by the family from 8 months because of its unpleasant taste; subsequently a (slight) increase in serum C5 value was noted. Individual serum free carnitine (C0) and C5 values in SBCADD patients are presented in Supplementary Figure 2.

**Figure 2:**
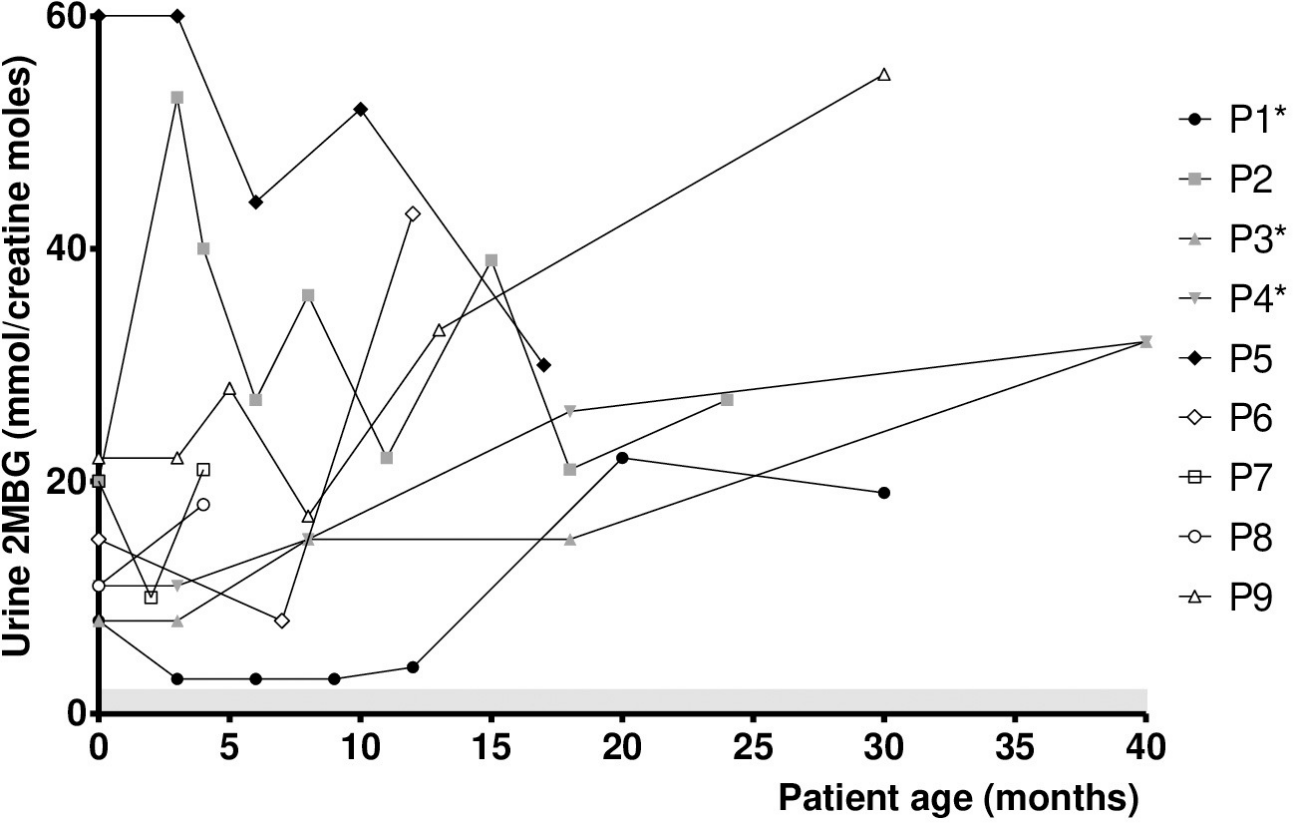
Urine 2MBG values in SBCADD patients. For each subject the first time point shows the urine 2MBG value detected upon NBS recall before starting the L-carnitine treatment (100 mg/kg/day). Subsequent time points show urine 2MBG value after starting with L-carnitine treatment. 2MBG reference range is highlighted (shaded area). *Data for P1 at 24 months, P3 and P4 at 12 and 24 months were not collected due to COVID19 pandemics.

Figure 2 presents the individual urine 2MBG values in SBCADD patients during the follow up. Urine 2MBG values stayed above the reference values in all subjects. In 7/9 SBCADD patients (P1-P4, P6, P8, P9) the individual urine 2MBG value increased (range +13/+119%). In 2/9 SBCADD patients (P5, P7) the individual urine 2MBG value decreased (−22%). A moderately variable trend in urine 2MBG values was noticed in all subjects. Urine 2MBG values in P3 and P4 (twin subjects) widely overlapped. No relationship between urine 2MBG values and L-carnitine intake, dietary information, or medical history could be noticed in 7/9 SBCADD patients (P1–P5, P7, P8). In P5 no major increase in urine 2MBG value occurred at 8 months, at the time when an increase in serum C5 value was noticed (see Figure 1). Conversely, an increase in urine 2MBG values was found in P6 at 12 months, after L-carnitine discontinuation. A (slight) increases in urine 2MBG values was also noticed in P9 after oral L-carnitine supplementation was discontinued.

In addition to 9 SBCADD patients, one subject (P10) was also enrolled who displayed increased C5 value on NBS DBS (0.45 μmol/L, reference 0.02–0.26) and increased urine 2MBG (15 mmol/creatinine moles, reference <2 mmol/) but could not be conclusively diagnosed with SBCADD. This subject was monitored for 7 months displaying both serum C5 and urine 2MBG values above the reference values. Such values were lower than median values found in SBCADD patients (mean serum C5: 0.85 μmoles/l; mean urine 2MBG: 12.0 mmol/creatinine moles).

## Discussion

SBCADD is an inherited disorder of amino acid metabolism in which L-isoleucine degradation is impaired (1). Due to the limited number of reported patients, its clinical relevance remains uncertain (13). Based on literature data 90% of SBCADD patients are asymptomatic (23). As most of these patients were ascertained by NBS, the inclusion of SBCADD in NBS programs is controversial (22). Nonetheless, there exist a group of SBCADD patients who develop (severe) health problems (6–8, 10, 11, 14). The reason why these patients become symptomatic remains unclear. Researchers speculate that some features, such as lethargy and muscle weakness, occur because L-isoleucine cannot be properly used for energy production in SBCADD (14). In addition, the enzyme defect may result in the accumulation of toxic compounds in the brain (24). Currently, neither recognized long-term monitoring biomarkers nor conclusive data on the efficacy of proposed treatments (i.e., L-carnitine supplementation, dietary protein restriction) are available for SBCADD.

In this study longitudinal biochemical data were systematically collected in SBCADD patients to define the applicability of currently employed diagnostic biomarkers as monitoring biomarkers. Particularly, this is the first study to present >12-month follow-up data on both serum C5 and urine 2MBG values in SBCADD patients. All enrolled subjects were diagnosed by NBS, allowing the first estimation of the incidence of SBCADD within Europe (1:17,780 newborns). Previous studies reported an incidence between 1:132 in the Hmong population (12) and 1:540,780 in non-Hmong groups (12, 25), indicating wide regional and ethnic differences.

Although serum C5 values stayed above the reference range in all subjects, a relative stabilization was observed in 7/9 SBCADD patients after starting oral L-carnitine supplementation, even with no dietary protein restriction. An increase in serum C5 values occurred in 2 SBCADD patients upon L-carnitine discontinuation (P6) and intercurrent illness (P5), respectively (Figure 1). Urine 2MBG values showed an inconsistent trend with larger variability among (and within) patients; no apparent relation with medical data could be found except in 2 patients (P6, P9) in whom L-carnitine discontinuation was followed by an increase in urine 2MBG excretion (Figure 2). Although neither concurrent increase in dietary protein intake nor clinical symptoms were detected, temporary subclinical (protein) catabolism cannot be ruled out in these 2 patients. Collective data suggest a possible role for serum C5 in patient (treatment) monitoring and may support the benefit of L-carnitine supplementation in SBCADD. Conversely, the role of urine 2MBG in patient monitoring remains unclear.

Few previous studies have presented biochemical data collected over time in SBCADD patients. However, data were either limited to DBS and/or serum C5 (23, 26) or scarce and/or with no defined time-point evaluations or not comparable to one another (5, 13, 22, 27) and always with a shorter follow-up as compared to the present study. In one study the follow-up duration was not mentioned (6). One SBCADD patient with a 4-year follow-up has also been reported for whom neither serum C5 nor urine 2MBG values were available (16).

As none of the patients enrolled in the present study developed symptoms related to SBCADD, no association between biochemical parameters and clinical phenotype could be investigated. Indeed, most of the patients showed *ACADSB* variants which have been previously found in asymptomatic patients (5, 8, 26–28). However, some variants (e.g., c.443C>T, c.908G>C, and c.1102T>C) found in the present study have been formerly reported in symptomatic SBCADD patients (23). Although specific variants have been most commonly found in symptomatic patients, no clear genotype-phenotype correlation exist in SBCADD and its clinical course cannot be easily predicted based on the genotype only. In fact, two siblings carrying the same ACADSB genotype (i.e., c.443C>T/c.1145C>T) have been reported, who presented with different clinical phenotypes (namely one being symptomatic and the other being asymptomatic, respectively) (23). Although the homozygous c.443C>T *ACADSB* variant has been previously described in symptomatic patients (22), one of the patients included in the present study carried the same genotype and was asymptomatic. Whether this patient will develop symptoms in the future or upon metabolic stress conditions (e.g., intercurrent illness, fasting) is unknown. Also, whether early diagnosis (by NBS) and treatment could have prevented the occurrence of symptoms in this patient remains to be determined.

Although no clear genotype-clinical phenotype exists, an association between *ACADSB* variants and biochemical phenotype has been reported. Consistently with previous observations showing serum C5 values up to up to 3 μmol/l in SBCADD patients carrying the homozygous variant c.443C>T (5, 22), P5 displayed the highest serum C5 values in the present study (Figure 1). In addition, an overlapping trend in serum C5 and urine 2MBG values was observed between the two twin subjects carrying the same *ACDSB* variants (P3 and P4). As the mechanism underlying these observations remains unresolved, future investigation is warranted.

Besides 9 SBCADD patients, 1 subject was also enrolled in the present study who only carried one pathogenic variant in the *ACADSB* gene and could not be conclusively diagnosed with SBCADD. Although below the median values found in SBCADD patients, serum C5 and urine 2MBG values were above the reference range in this subject. Despite the short follow-up data, serum C5 values found in P10 were comparable to individual SBCADD patients. Thus, SBCADD could not be ruled out in this subject. Interestingly, in the present study serum C5 values were found between 0.5 and 2.8 μmol/l in SBCADD patients and 0.8-0.9 μmol/l in the subject with no conclusive diagnosis of SBCADD. We speculate about a potential role of serum C5 values in subjects' stratification. In principle, a possible role of serum C5 value in clarifying the (biochemical) impact of (novel) *ACADSB* variants may be hypothesized. On the other hand, the circumstance of increased urine 2MBG excretion in conditions other than SBCADD appears intriguing. Future studies addressing these issues are worthy.

Potential limitations of this study include the retrospective design and the absence of functional studies. In addition, it is unknown whether higher L-carnitine dose and/or dietary protein restriction could have resulted in normalization of serum C5 and/or urine 2MBG values in the enrolled subjects. No direct correlation between serum C0 and C5 values was found in the present study. Indeed, P1 and P5 showed stable serum C5 values despite displaying supraphysiological serum C0 values at several time points (Figure 1). Yet, it cannot be ruled out that supraphysiological serum C0 values may have concurred to C5 accumulation in P2 (Supplementary Figure 2). Conversely, P6 and P9 displayed a decreasing trend in serum C0 levels after L-carnitine supplementation discontinuation. Although all patients showed (supra)normal serum C0 values in the present study, longer follow-up is required to assess whether untreated SBCADD patients are at risk of developing carnitine deficiency. Whether serum C5 and urine 2MBG values accurately reflected their tissue concentrations in SBCADD also remains to be ascertained.

In conclusion, the clinical course of SBCADD remains poorly predictable. Therefore, regular clinical monitoring (including serum glucose, ammonia and CK concentrations, liver function tests, blood gases, heart ultrasound, electrocardiogram, weight, height, and psychomotor development assessment) together with general advice on how to prevent catabolism is recommended (15, 23, 29). As serum C5 appears as a potential long-term biomarker for patients' (treatment) monitoring we suggest including regular serum C5 assessment in patients' monitoring. Conversely, available data do not support a role for urine 2MBG beyond the diagnosis. Although previous untreated cases have been reported that remained asymptomatic over a variable period of time (5, 14, 26), L-carnitine supplementation may be beneficial (at least) on the “biochemical phenotype” in SBCADD. Yet, appropriate dose titration is recommended in order to avoid supraphysiological free carnitine levels potentially concurring to C5 accumulation. The clinical relevance of such “biochemical benefit” remains unclear. On the other hand, the role of dietary protein restriction in SBCADD remains to be established. Longer follow-up studies are warranted to identify accurate monitoring (and prognostic) biomarkers and define the optimal management approach in SBCADD.

## Data availability statement

The raw data supporting the conclusions of this article are available from the corresponding author upon reasonable request.

## Ethics statement

The studies involving human participants were reviewed and approved by the Medical Ethical Committee of the University of Naples “Federico II”. Written informed consent from the participants' legal guardian/next of kin was not required to participate in this study in accordance with the national legislation and the institutional requirements.

## Author contributions

AR and MT wrote the first version of the manuscript. GF, GV, PS, GP, and MR critically reviewed the manuscript. All authors substantially contributed to the work and were involved in conception and design of the study and/or analysis and interpretation of data, and revising the article critically for important intellectual content. All authors approved the final manuscript as submitted and agree to be accountable for all aspects of the work. All authors confirm the absence of previous similar or simultaneous publications.

## Conflict of interest

The authors declare that the research was conducted in the absence of any commercial or financial relationships that could be construed as a potential conflict of interest.

## Publisher's note

All claims expressed in this article are solely those of the authors and do not necessarily represent those of their affiliated organizations, or those of the publisher, the editors and the reviewers. Any product that may be evaluated in this article, or claim that may be made by its manufacturer, is not guaranteed or endorsed by the publisher.

## Supplementary material

Supplementary Figure 1 Regional Operative Procedure for newborns suspected with an inherited metabolic disease at newborn screening.

Supplementary Figure 2 Individual serum free carnitine (C0, black circles, top section of the graph) and 2-methylbutyryl carnitine (C5, gray squares, bottom section of the graph) values in SBCADD patients. For each subject the first time point shows the serum C5 and C0 values detected before starting the L-carnitine treatment. Subsequent time points show serum C5 value after starting with L-carnitine treatment, unless stated otherwise. C0 reference range (10–45 μmol/L) is shown (dashed lines). ^#^Samples collected after carnitine discontinuation.
