# Supplementary material for: Long-term monitoring for short/branched-chain acyl-CoA dehydrogenase deficiency: A single-center 4-year experience and open issues
Source: Front Pediatr. 2022 Sep 6;10:895921. doi: 10.3389/fped.2022.895921 (PMC9485620; doi:10.3389/fped.2022.895921)
Supplement: Supplementary Figure 1 — Regional Operative Procedure for newborns suspected with an inherited metabolic disease at newborn screening. [file Table_1.DOCX]

YES

YES

YES

SPOT BLOOD SAMPLING between the 48^th^ and the 72^th^ hour of newborn’s life

DELIVERY OF THE SAMPLE TO THE COURIER

WAS SPOT BLOOD DONE CORRECTLY?

REQUEST FOR NEW SAMPLE AT THE BIRTH CENTER

SCREENING REPORT are sent to newborn centres for negative and positive cases

SAMPLE ACCEPTANCE

NEWBORN RECALL AND NEW SPOT BLOOD COLLECTION

SPOT ANALYSIS

POSITIVITY TO ANALYTES?

EMERGENCY SITUATION?

DIRECT NEWBORN RECALL FROM THE CLINICAL CENTER

Ì

NO

NO

NO

DIAGNOSTIC TESTS OF 2ND LEVEL

POSITIVITY TO ANALYTES?

NO

MOLECULAR ANALYSIS (on peripheral blood sample)

YES

**Supplemental figure 1.**

YES

YES

YES
