# Supplementary material for: Long-term monitoring for short/branched-chain acyl-CoA dehydrogenase deficiency: A single-center 4-year experience and open issues
Source: Front Pediatr. 2022 Sep 6;10:895921. doi: 10.3389/fped.2022.895921 (PMC9485620; doi:10.3389/fped.2022.895921)
Supplement: Supplementary Figure 2 — Individual serum free carnitine (C0, black circles, top section of the graph) and 2-methylbutyryl carnitine (C5, gray squares, bottom section of the graph) values in SBCADD patients. For each subject the first time point shows the serum C5 and C0 values detected before starting the L-carnitine treatment. Subsequent time points show serum C5 value after starting with L-carnitine treatment, unless stated otherwise. C0 reference range (10–45 μmol/L) is shown (dashed lines). #Samples collected after carnitine discontinuation. [file Table_2.DOCX]

**Supplemental figure 2.**
